# Performance of Valencia sweet orange grafted onto dwarfing citrandarins

**DOI:** 10.3389/fpls.2025.1530396

**Published:** 2025-03-06

**Authors:** Fernando Trevizan Devite, Marinês Bastianel, Mariângela Cristofani-Yaly, Ana Júlia Borim de Souza, Biana Pelissari Gadanhoto, Ana Carolina Costa Arantes, Fernando Alves De Azevedo

**Affiliations:** Sylvio Moreira Citrus Center, Agronomic Institute, Cordeirópolis, São Paulo, Brazil

**Keywords:** citrus hybrids, rootstock diversification, fruit yield, hydric stress, *Candidatus Liberibacter asiaticus*

## Abstract

Rootstock diversification is increasingly desired and necessary for the sustainability of citriculture, which is subject to adversity, such as the occurrence of Huanglongbing (HLB), which has impacted activity in most producing regions of the world. The objective was to evaluate the productivity, water-use efficiency, vegetative growth, and HLB incidence of Valencia sweet orange grafted onto three citrandarins (dwarfing) and Swingle citrumelo (standard). The field experiment was conducted under a high-density planting spacing of 5.0 m × 1.5 m (1333 plants ha^−1^) for citrandarin rootstocks (IAC 1600, IAC 1697, and IAC 1711) and a conventional spacing of 6.8 m × 2.5 m (588 plants ha^−1^) for Swingle citrumelo. The Swingle citrumelo rootstock combination with Valencia orange exhibited a larger canopy volume and higher per-plant yield, demonstrating high productivity under conditions of adequate water availability and conventional spacing. However, this combination also demonstrated lower water-use efficiency and higher susceptibility to HLB, particularly in 2024, highlighting its limitations for use in HLB-endemic regions. In contrast, combinations with citrandarins, especially IAC 1600, showed greater water-use efficiency, smaller canopy volume, and increased drought tolerance in higher-density planting. These combinations were also less susceptible to HLB and performed well in high-density planting systems, promoting the productive efficiency (kg fruit m^3^ canopy). The Swingle combination is recommended to maximize production in areas with abundant water resources and low HLB pressure, while citrandarin combinations, particularly IAC 1600, are better suited for regions with limited water availability and high HLB incidence.

## 1 Introduction

Sweet orange (*Citrus × sinensis* L. Osbeck) is the most extensively cultivated citrus species globally, originating from Asia, with northeastern India and northern Burma recognized as its center of domestication (Seminara et al., 2023). This crop plays a critical role in the global fruit industry, with Brazil leading production at 16.9 million tons harvested from 568,132 ha, followed by India, China, and Mexico (FAOSTAT, 2024). In Brazil, São Paulo and the Triângulo Mineiro/Southwestern Minas Gerais, the so-called citrus belt, account for 83.8% of national production, underscoring their strategic importance to the agricultural economy (Fundecitrus, 2024; IBGE, 2024).

Rootstocks are indispensable in citriculture, as they determine the tree's vigor, architecture, nutrient uptake, yield potential, and resistance to biotic and abiotic stressors (Cristofani-Yaly et al., 2007; Castle, 2010; Carvalho et al., 2019). The scion-rootstock interaction further influences key fruit attributes such as size, peel thickness, juice quality, and shelf life, which directly affect marketability (Pompeu Junior, 2005). Advanced rootstocks such as Carrizo citrange and US-942, have shown superior performance under stress conditions, enhancing fruit yield and quality while providing increased resilience against diseases, such as Huanglongbing (HLB) (Albrecht and Bowman, 2012; Singerman et al., 2021). Moreover, high-density planting systems incorporating size-controlling rootstocks, such as tetraploid hybrids, have optimized production efficiency and disease management in citriculture (Grosser et al., 2015).

Despite these advancements, the Brazilian citrus industry remains heavily dependent on a narrow genetic base of rootstocks, with Rangpur lime (*Citrus × limonia* Osbeck) and Swingle citrumelo (*Citrus × paradisi* Macfad. × *Poncirus trifoliata*) accounting for approximately 80% of seedlings produced in nurseries (Carvalho et al., 2019). This limited genetic diversity increases the sector's vulnerability to phytosanitary challenges, such as Citrus tristeza virus (CTV), citrus canker, and Huanglongbing (HLB). Therefore, rootstock diversification is essential for the sustainability of citriculture, particularly in the face of climate change and the rising incidence of diseases like HLB, which pose significant threats to global citrus production.

Huanglongbing (HLB), or citrus greening disease, is among the most destructive threats to citrus production worldwide. The disease is caused by three bacterial species—*Candidatus Liberibacter asiaticus* (CLas), *Candidatus Liberibacter africanus* (CLaf), and *Candidatus Liberibacter americanus* (CLam)—transmitted by the Asian citrus psyllid (*Diaphorina citri* Kuwayama) (Bové, 2006). CLas is the dominant species in symptomatic plants in São Paulo, Brazil, where HLB was first reported in 2004 (Coletta Filho et al., 2004). By 2024, the disease had affected 44.3% of trees in the citrus belt, equivalent to 90.4 million plants (Fundecitrus, 2024), emphasizing the urgent need for effective management.

At the time of its initial detection in Brazil, the incidence of HLB was relatively low compared to regions where the disease was already widespread and caused severe economic losses, such as Florida. This provided Brazilian researchers and citrus growers with a critical window of opportunity to implement management strategies aimed at delaying disease establishment and minimizing its impact. Early interventions included planting pathogen-free nursery stock, systematically removing of symptomatic trees, and employing chemical and biological vector control measures (Belasque et al., 2010; Bassanezi et al., 2011). These proactive efforts enabled for a more measured response compared to the reactive strategies necessitated in areas, where HLB had already reached epidemic proportions, such as Florida.

Rootstocks significantly influence HLB severity, as vigorous growth encourages psyllid feeding, accelerating disease spread (Pompeu Junior, 2005; Halbert and Manjunath, 2004). Conversely, less vigorous rootstocks limit shoot production and suppress psyllid populations (Hall et al., 2013; Stover et al., 2016). These insights guide rootstock selection in Brazil, where less vigorous options are part of integrated HLB management strategies (Bassanezi et al., 2011).

As HLB incidence rises in Brazil, integrated strategies combining rootstock selection with cultural practices and vector control have become increasingly critical (Hall et al., 2013; Sétamou et al., 2016). Dwarfing rootstocks, such as Flying Dragon (*C. trifoliata* var. *monstrosa* T. Ito), show promise in reducing canopy volume and psyllid feeding sites, and enhancing disease control (Rodrigues et al., 2020). Continued research into rootstock-scion interactions and integrated pest management is vital to strengthening citrus production resilience in HLB-affected regions.

Citrandarins are highly valued as citrus rootstocks for their unique combination of disease resistance and agronomic benefits. These rootstocks provide tolerance to citrus decline and Exocortis viroid, immunity to CTV, and resistance to citrus nematodes (*Tylenchulus semipenetrans*) and *Phytophthora* gummosis, which are major threats to citrus productivity (Blumer and Pompeu Junior, 2005; Schinor et al., 2013). Furthermore, citrandarins enhance cold tolerance and promote compact, highly productive plants, making them integral to sustainable citrus production across diverse environments (Blumer and Pompeu Junior, 2014).

Recent studies have confirmed the horticultural advantages of citrandarins, emphasizing their potential for seedling production and high-density planting systems, which significantly improve orchard productivity (Schinor et al., 2013; Rodrigues et al., 2015; Conceição et al., 2019). High-density planting facilitates cultural practices and harvesting, while also serving as an effective strategy to mitigate losses caused by diseases such as HLB. This approach allows for a greater number of plants per unit area, leading to enhanced productivity (Cantuarias-Avilés et al., 2012).

According to data from Coordenadoria de Defesa Agropecuária of the SP State/SSP (2023), Swingle accounted for 56.9% of the rootstocks in seedlings sold in 2023, followed by Rangpur lime at 17.9% and IAC 1710 and Indio citrandarins, at 10.0% and 5.9%, respectively, indicating a significant increase in the use of citrandarins. The combined share of citrandarins now surpasses that of the Rangpur lime rootstock. Despite this progress, comprehensive evaluations of their performance in high-density planting systems under HLB management are lacking. Such investigations are crucial for optimizing productivity and addressing the challenges posed by climate variability and disease pressure.

This study aimed to evaluate the agronomic performance of Valencia sweet orange grafted onto three citrandarin rootstocks (IAC 1600, IAC 1697, and IAC 1711) under high-density planting conditions. By integrating advanced rootstock technology with sustainable management practices, this research seeks to provide actionable insights for enhancing the resilience and competitiveness of Brazilian citriculture in a rapidly changing agricultural landscape.

## 2 Materials and methods

**Figure 1:**
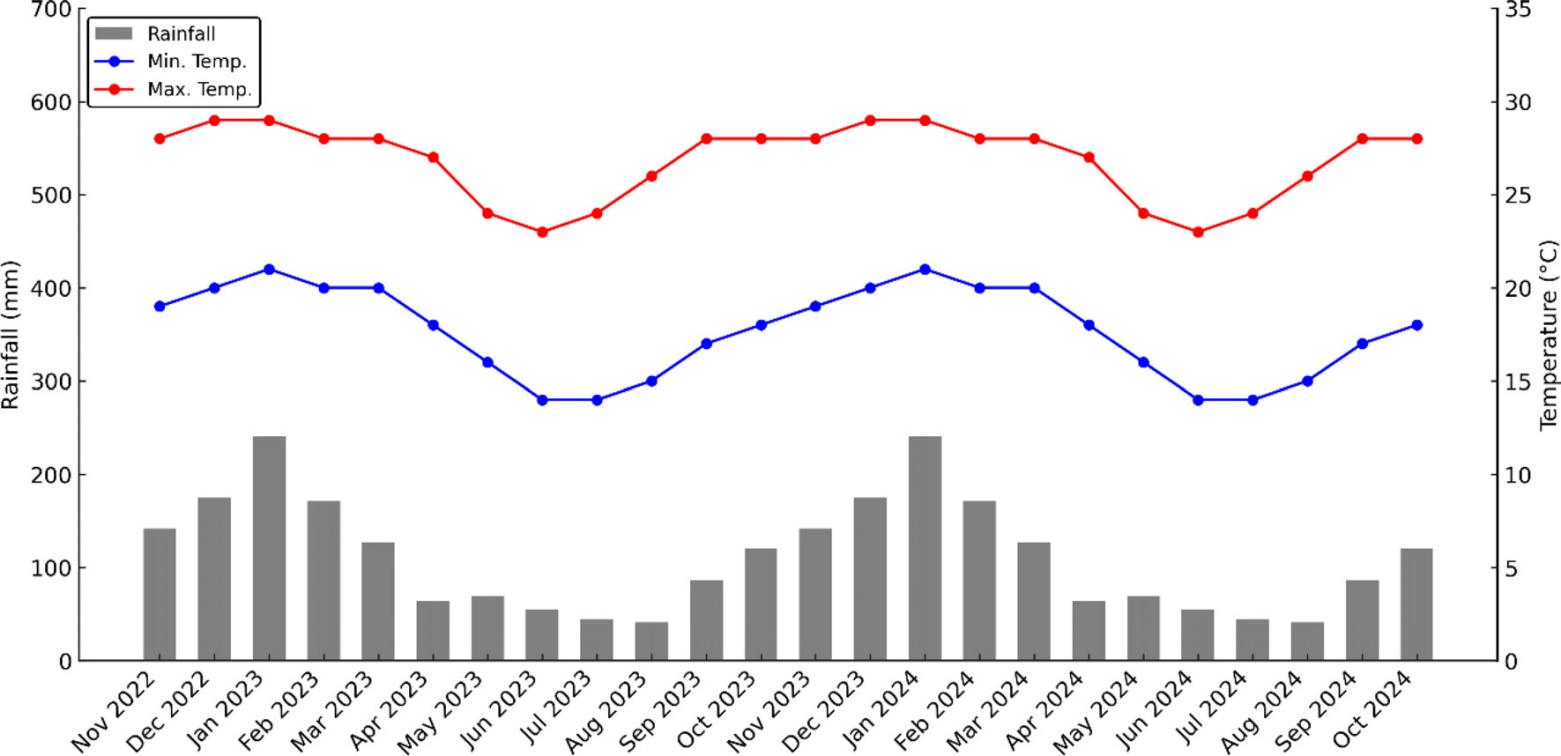
Monthly average values of minimum (Min. Temp.) and maximum temperatures (Max. Temp.) and monthly accumulated rainfall during the bearing period of the experiment (2022–2024) (Santa Cruz do Rio Pardo/SP).

The experiment was established in 2017 at the Agroterenas Groups commercial farm in Santa Cruz do Rio Pardo, Sao Paulo State, Brazil, located at the geographic coordinates 22°48’53.4”S 49°22’50.9”W and at an altitude of 467 m. The region’s predominant climate is classified as Cwa (humid subtropical with hot summers and dry winters) according to Köppen's classification. The soil was classified as Red Latosol, with a high drainage capacity and good depth. Annual average temperature and rainfall data (**Figure 1**). Based on climatological records collected between November 2022 and October 2024, the average annual precipitation was approximately 1,335 mm, and the average maximum and minimum temperatures were around 26.8 and 17.7°C, respectively. These data were obtained monthly and represent monthly averages of temperature and precipitation for the analyzed period.

Without irrigation, Valencia sweet orange plants were grafted onto three citrandarins, namely: IAC 1600 or X-639 (*Citrus reshni* hort. ex Tanaka ‘Cleopatra’ × *Poncirus trifoliata* (L.) Raf. ‘Rubidoux’), IAC 1697 or US-812 (*C. sunki* hort. ex Tanaka ‘Sunki’ × *P. trifoliata* (L.) Raf. ‘Benecke’), and IAC 1711 or US-815 (*C. reticulata* Blanco ‘Changsha’ x *P. trifoliata* (L.) Raf. ‘English Large’), produced by the United States Department of Agriculture, Florida, USA, and introduced to the Sylvio Moreira Citrus Center, Cordeirópolis, São Paulo State, Brazil, in 1982 (Blumer, 2005). The plants were spaced at 5.0 m × 1.5 m (1,333 plants per ha), with Swingle citrumelo as the standard rootstock spaced at 6.8 m × 2.5 m (588 plants per ha).

Annual fertilization was conducted according to the recommendations of Mattos Junior et al. (2022), using a balanced NPK fertilizer applied in three split doses during the growing season. Psyllid and other pest control were conducted using specific foliar insecticides, such as neonicotinoids and pyrethroids, rotating chemical groups at intervals of 15 - 20 days, occasionally extending to 30 days to prevent resistance development.

### 2.1 Drought tolerance

During the winters of 2023 and 2024, visual assessments of leaf rolling, a physiological indicator of water deficit, were conducted. Evaluations were carried out at the peak of the seasonal water stress period, examining eight plants per plot for each rootstock combination. Plants were scored on a standardized scale from 1 to 3, following the methodology described by Schinor et al. (2013). The scoring criteria were defined as follows: (1) severe leaf rolling, characterized by all leaves showing pronounced rolling, potentially accompanied by dryness; (2) moderate leaf rolling, with leaves exhibiting slight rolling; and (3) no leaf rolling, with all leaves maintaining a normal blade structure.

The leaf water potential was assessed following the protocol described by Kaufmann (1968) as a complementary analysis to the visual evaluation of drought tolerance. Leaves were sampled from eight plants per plot for each scion/rootstock combination, targeting the middle third of the canopy before sunrise, in accordance with the guidelines of MaChado et al. (2002). Immediately after collection, each leaf was placed in an individual hermetically sealed bag, stored in an ice-filled cooler, and transported to the laboratory for evaluation. The measurements were conducted using a Scholander-type pressure chamber (PMS Instrument, Model 1000, Corvallis, USA), and the results were expressed in megapascals (MPa).

### 2.2 Leaf gas exchange analysis

Leaf gas exchange analyses were conducted using an Infrared Gas Analyzer (IRGA), model LCpro (ADC, Hoddesdon, UK). Measurements were performed in winter 2023, with 8 plants per plot for each scion–rootstock combination. The variables assessed included stomatal conductance (gs, mol m^-2^ s^-1^), CO_2_ assimilation rate (A, μmol m^-2^ s^-1^), transpiration (E, mmol H_2_O m^-2^ s^-1^), and internal CO_2_ concentration in the substomatal cavity (Ci, mol m^-2^ s^-1^).

### 2.3 Vegetative development and fruit yield

Vegetative development was assessed through precise measurements of plant height and scion diameter in 8 plants per plot for each scion–rootstock combination. Plant height was measured using a graduated ruler aligned parallel to the geotropic growth axis of the canopy, and scion diameter was measured parallel to the soil surface at a height of 1.5 m. Following Mendel (1956), the canopy volume (V) was calculated using the equation $\;\text{V}=\frac{2}{3}\times \text{π}\times {\text{R}}^{2}\times \text{V}$ , where V represents the canopy volume in cubic meters, R is canopy radius, and H is scion height.

Annual yield was evaluated during the 2022–2024 harvest seasons by weighing the total harvested fruits from 8 plants per plot for each scion–rootstock combination, providing the average fruit yield per plant in kilograms. Harvests were conducted once the fruit reached optimal visual maturity. Production efficiency was calculated as the ratio of yield (kg of fruit per plant) to canopy volume (m³), expressed as kg of fruit per m³.

### 2.4 Manual harvest efficiency

During the 2023 and 2024 harvest seasons, the efficiency of manual harvesting across different rootstock combinations was evaluated by measuring the time required to harvest fruit. The assessment was conducted in three blocks, each containing five plants of a given rootstock combination. Trained pickers, who typically harvest an average of 80 boxes per day, carried out the task on the same day to ensure consistency. The harvest time for each plant was recorded using a stopwatch, measuring the time taken by a single picker to collect fruit from five trees per block. The results were expressed as the number of boxes harvested per hour per worker, providing a standardized metric for manual harvesting efficiency.

### 2.5 Physicochemical fruit evaluations

Fruit quality assessments were conducted during the 2022–2024 seasons using the same trees evaluated for fruit yield. For each rootstock combination, 5 trees per block (4 blocks) were selected in a randomized block design, and 20 fruits were sampled. Sampling was based on visual criteria, including fruit size and color, with fruits harvested from the median height of the canopy across all four quadrants of the scion. Sampling was based on the visual characteristics of fruit size and color, with the fruits collected from the median height of the four quadrants of the scion. The samples were then sent to the Industrial Laboratory at Agroterenas (Santa Cruz do Rio Pardo/SP). The physicochemical quality of the fruits was evaluated using the following variables: weight (g); juice content (%); soluble solids content (SS) determined with a direct reading refractometer at 20°C (°Brix); and titratable acidity (TA) obtained by titration with 0.3125 N sodium hydroxide and expressed as a percentage of citric acid per 100 mL of juice (%). The ratio was calculated by the arithmetic ratio of SS to TA, and the technological index (TI) was determined by the following equation: TI = juice content(%)×SS × mass of the standard industrial citrus box.

### 2.6 Fruit maturation curve

The SS, TA, and the ratio (SS/TA) were analyzed as a function of days before harvest (DBH), using regression models to identify variation patterns over time. In the 2023 season, fruit evaluations began on September 13, with data collected biweekly until the harvest on October 27. In the 2024 season, due to severe drought and premature fruit drop, evaluations were initiated earlier, on August 20, and conducted every 10 days until harvest on September 20. These adjustments ensured a more precise analysis of the impact of adverse climatic conditions on fruit quality parameters during the pre-harvest period. For each evaluation, 20 fruits were analyzed per sample (four collection dates before harvest), collected from five plants within each block. The experiment followed a randomized block design with four blocks per rootstock combination.

### 2.7 Huanglongbing incidence

From 2022 to 2024, trees were inspected annually to diagnose the visual symptoms of HLB, aiming to determine disease incidence in each rootstock combination. This measure was based on the cumulative number of symptomatic trees relative to the initial number of plants. Inspections were conducted by two continuously trained inspectors skilled in identifying trees with HLB symptoms. The cumulative incidence values for each year were used to calculate the area under the disease progress curve (AUDPC), following the equation proposed by Shaner and Finney (1977).

### 2.8 Statistical analyses

Data were subjected to normality analysis, analysis of variance (ANOVA), and multiple comparison tests using Tukey tests (α = 0.05). The tests were performed on RStudio software (v. 4.1.0). Multivariate analyses using principal component analysis (PCA) were carried out using a correlation matrix to identify citrus group responses. PCA data were normalized and displayed in a biplot of principal component distances from their mean values (Zuur et al., 2007). PCA was conducted using OriginPro software (8.6), and mean separation tests were performed using the agricolae package (Mendiburu, 2021).

## 3 Results and discussion

### 3.1 Drought tolerance

**Figure 2:**
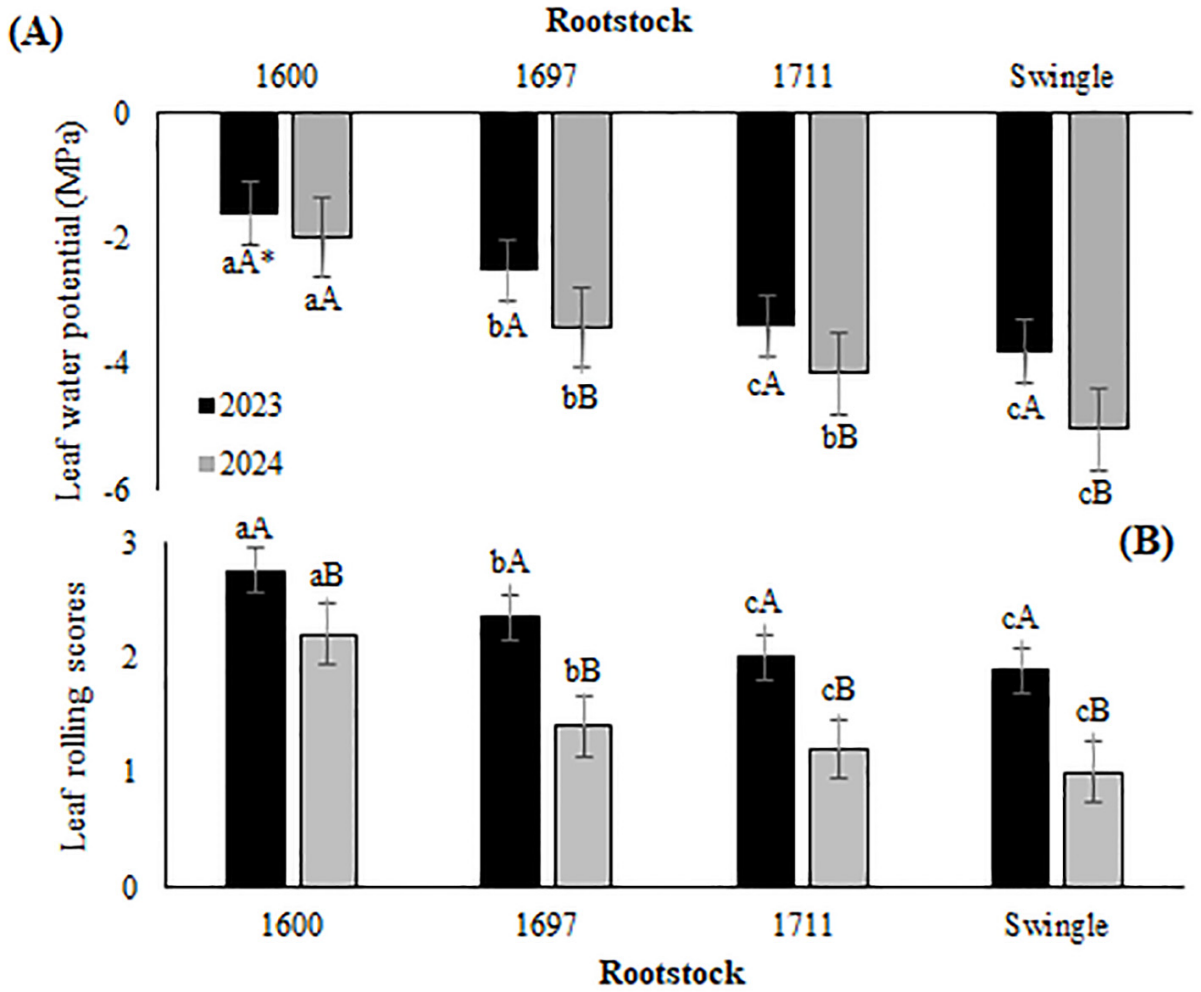
**(A)** Leaf water potential (MPa) across different rootstocks; **(B)** Leaf rolling scores in Valencia orange grafted onto three citrandarins (IAC 1600, IAC 1697, and IAC 1711) and Swingle citrumelo in Santa Cruz do Rio Pardo (2023–2024). *Means followed by the same lowercase letter among rootstocks within the same year and uppercase letters within the same rootstock across years indicate no significant differences according to Tukey's test (p < 0.05).

The water stress analyses conducted during the winters of 2023 and 2024 (**Figures 2A, B**) revealed significant differences in drought tolerance among the rootstocks studied. Valencia sweet orange grafted onto the IAC 1600 citrandarin exhibited superior drought tolerance, as indicated by higher leaf water potential values (−1.6 and −1.98 MPa) and greater average leaf rolling scores (2.75 and 2.2) across both years. This combination effectively maintained higher water potential, even under significant water deficit conditions, highlighting its resilience.

In 2024, when water stress was more severe, trees grafted onto citrandarin rootstocks demonstrated a better physiological response, likely due to enhanced osmotic adjustment and stomatal regulation, compared to those grafted onto Swingle citrumelo, which exhibited pronounced leaf rolling and higher stress levels under similar conditions.

Rootstocks exhibiting greater drought tolerance, such as IAC 1600 citrandarin, showed enhanced osmotic adjustment capabilities. These mechanisms enabled the maintenance of a higher leaf water potential during drought by preserving cellular turgor and sustaining photosynthetic activity. According to Silva et al. (2019), trifoliate rootstocks induce osmotic adjustments and cell wall hardening, traits that are incorporated into citrandarins through genetic improvement programs involving crosses with drought-resistant varieties. These inherited traits enhance the ability of citrandarins to sustain physiological functions under water stress, ensuring the survival and productivity of grafted plants.

The interplay between leaf water potential and stomatal regulation is critical for drought resilience in citrus plants, particularly citrandarins. These rootstocks exhibit precise stomatal control, reducing water loss through transpiration while maintaining adequate water potential. This balance allows essential processes, such as CO_2_ assimilation, to continue while minimizing hydraulic failure. By initiating early stomatal closure in response to declining water potential, citrandarins reduce the risk of xylem cavitation, preserve hydraulic conductivity, and enhance the drought tolerance of the scion (Rodriguez-Gamir et al., 2010).

Additionally, the anatomical adaptations of citrandarins contribute significantly to drought resistance. Similar to observations by Shafqat et al. (2021) in sour orange, Valencia sweet orange grafted onto citrandarins exhibited traits such as thicker epidermal cells and xylem, which enhance resistance to water flow and improved nutrient transport efficiency. Other features, including thicker pit membranes and increased embolism resistance—commonly observed in rootstocks such as Rangpur lime and Swingle citrumelo (Miranda et al., 2020)—were also evident in citrandarins, supporting a conservative water-use strategy. These adaptations enable citrandarins to respond effectively to reduced water availability by minimizing water loss and mitigating stress-related damage. Consequently, the evaluated citrandarins demonstrated varying levels of water-use efficiency and drought resilience.

### 3.2 Leaf gas exchange analysis

**Figure 3:**
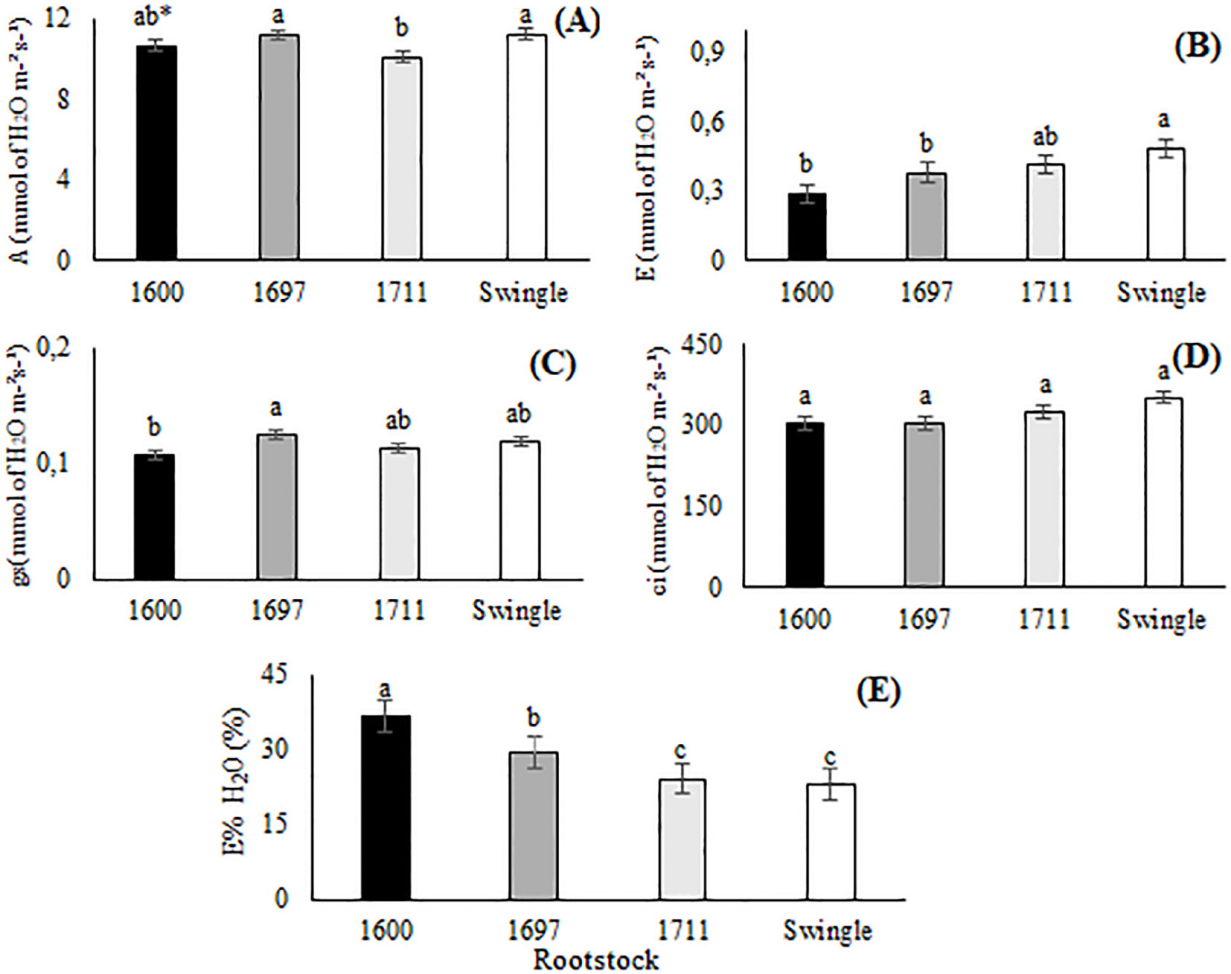
**(A)** - CO_2_ assimilation (A); **(B)** - Transpiration (E); **(C)** - Stomatal conductance (gs); **(D)** - Intercellular CO_2_ concentration (ci); and **(E)** - Water-use efficiency (E%H_2_O) in Valencia sweet orange grafted onto three citrandarins (IAC 1600, IAC 1697, and IAC 1711) and Swingle citrumelo (Santa Cruz do Rio Pardo, Brazil, 2023). Means followed by the same letter do not differ according to Tukey's test (p<0.05).

The results for CO_2_ assimilation rate (A) demonstrated that Swingle citrumelo and IAC 1697 citrandarin induced the highest values in Valencia sweet orange, at 11.23 and 11.19 μmol m^-2^ s^-1^, respectively (**Figure 3A**). In contrast, the combination with IAC 1711 citrandarin exhibited the lowest assimilation rate, at 10.11 μmol m^-2^ s^-1^. Regarding transpiration (E), Swingle citrumelo showed the highest rate (0.48 mmol H_2_O m^-2^ s^-1^), while IAC 1600 citrandarin exhibited the lowest rate (0.29 mmol H_2_O m^-2^ s^-1^), indicating a potential advantage in water-use efficiency for the latter (**Figure 3B**).

Stomatal conductance (gs) varied among rootstock combinations, with IAC 1600 citrandarin displaying the lowest value (0.10 mol m^-2^ s^-1^) and Swingle citrumelo displaying the highest (0.12 mol m^-2^ s^-1^) (**Figure 3C**). Intercellular CO_2_ concentration (ci) remained consistent across rootstocks, indicating no significant genetic influence on CO_2_ diffusion into leaves (**Figure 3D**). Water-use efficiency (E%H_2_O) was highest in the IAC 1600 citrandarin combination at 36.77%, followed by IAC 1697 citrandarin at 29.56%. Swingle citrumelo and IAC 1711 citrandarin exhibited lower efficiencies, at 24.22 and 23.09%, respectively (**Figure 3E**).

The high CO_2_ assimilation rates observed in the Swingle citrumelo and IAC 1697 citrandarin combinations highlight their superior photosynthetic capacity, likely driven by favorable genetic traits and environmental adaptability, as previously reported by Miranda et al. (2020). The study's climatic data, with average maximum temperatures of 26.8°C and minimum temperatures of 17.7°C, coupled with seasonal rainfall patterns, influenced photosynthesis and transpiration rates, as well as water-use efficiency. As observed by Miranda et al. (2020), rootstock-mediated stomatal regulation and water redistribution capacity play a significant role in optimizing gas exchange processes under varying environmental conditions, which aligns with the elevated transpiration and CO_2_ assimilation rates observed in the Swingle citrumelo and IAC 1697 combinations.

Transpiration rates (E) were highest in the Swingle citrumelo and IAC 1711 citrandarin combinations, suggesting greater water loss through gas exchange. Although this can facilitate CO_2_ uptake, it may also reduce water-use efficiency under drought stress. Miranda et al. (2020) reported that Swingle citrumelo enhanced the water redistribution capacity, which could explain its elevated transpiration rate.

Stomatal conductance (gs) was lowest in the IAC 1600 citrandarin combination, a trait associated with reduced water loss and improved water-use efficiency — key advantages under water deficit conditions. This finding aligns with that of Mesquita et al. (2016), who demonstrated higher stomatal conductance in plants grafted onto Swingle citrumelo, further supporting its utility in regions frequently exposed to water stress (Sanchez et al., 2006).

The consistency in intercellular CO_2_ concentration (ci) among rootstocks suggests that genetic differences had little impact on CO_2_ diffusion into the leaves. Instead, stomatal conductance and transpiration appear to play a more significant role in photosynthetic efficiency. Selecting rootstocks that optimize these factors is critical for enhancing citrus productivity (Papadakis et al., 2004).

Furthermore, osmotic adjustments and stomatal regulation, as observed in the IAC 1600 citrandarin combination, play a critical role in enhancing water-use efficiency. Santana-Vieira et al. (2016) highlighted the significance of these mechanisms in drought-resistant rootstocks, which enable sustained photosynthesis even under minimal water availability. This trait is especially beneficial for citrus cultivation in regions facing water scarcity. The superior water-use efficiency demonstrated by IAC 1600 suggests that this rootstock is a promising option for areas with limited water resources, thereby supporting the sustainability of citrus production.

### 3.3 Vegetative development and fruit yield

Swingle citrumelo induced vigorous scion growth (**Table 1**), producing the largest canopy volume, trunk diameter, and plant height among the evaluated rootstocks. This growth is partially attributed to the wider planting spacing (6.8 m × 2.5 m) used for Swingle compared to the higher-density configuration for citrandarins (5.0 m × 1.5 m), which allowed greater access to resources such as light, water, and nutrients (Blumer and Pompeu Junior, 2005). However, vigorous growth promotes intense vegetative flushing, providing abundant feeding and breeding sites for the Asian citrus psyllid (Schinor et al., 2020; Girardi et al., 2021). This likely explains why Swingle exhibited the highest HLB incidence in this study, highlighting its limitations in disease-endemic regions.

**Table 1:** Growth parameters of Valencia sweet orange trees grafted onto 3 citrandarin rootstocks (IAC 1600, IAC 1697, and IAC 1711) and Swingle citrumelo.

Rootstock	2022			2023			2024		
	H (m)	D (m)	V (m^3^)	H (m)	D (m)	V (m^3^)	H (m)	D (m)	V (m^3^)
IAC 1600	1.7 ± 0.10 b*	1.7 ± 0.18 c	2.7 ± 0.58 c	1.8 ± 0.03 b	1.8 ± 0.14 c	3.0 ± 0.56 c	1.8 ± 0.14 b	1.9 ± 0.05 c	3.5 ± 0.60 c
IAC 1697	2.0 ± 0.17 a	2.5 ± 0.16 b	7.4 ± 0.92 b	1.9 ± 0.11 b	2.4 ± 0.13 b	7.5 ± 0.52 b	2.2 ± 0.11 a	2.6 ± 0.08 b	7.6 ± 0.92 b
IAC 1711	2.3 ± 0.19 a	2.4 ± 0.15 b	7.2 ± 0.87 b	2.2 ± 0.05 a	2.4 ± 0.09 b	8.5 ± 0.57 b	2.2 ± 0.13 a	2.8 ± 0.11 b	10.3 ± 0.81 b
Swingle	2.3 ± 0.12 a	4.2 ± 0.24 a	21.6 ± 1.08 a	2.3 ± 0.08 a	4.5 ± 0.10 a	23.4 ± 2.07 a	2.4 ± 0.29 a	4.5 ± 0.15 a	26.0 ± 1.85 a
CV (%)	17.3	12.2	18.7	14.7	11.9	19.4	16.1	13.6	19.5

*Means followed by the same letter within each column do not differ by Tukey’s test (p<0.05).

Parameters include tree height (m), diameter (m), and canopy volume (m³). Data were collected from a rainfed orchard affected by Huanglongbing in Santa Cruz do Rio Pardo, São Paulo, Brazil, during the 2022–2024 evaluation period.

In contrast, citrandarin hybrids, particularly IAC 1600, displayed a compact growth habit with a smaller canopy volume, reduced trunk diameter, and shorter plant height, consistent with their dwarfing genetic traits (Schinor et al., 2020). These traits limit vegetative flushing, reducing psyllid attraction and HLB incidence. Additionally, IAC 1600 showed superior adaptability to high-density planting, optimizing productivity per unit area while demonstrating resilience to water stress and disease pressure. These findings align with Girardi et al. (2021), who highlighted the benefits of less vigorous rootstocks for managing HLB in intensive systems by minimizing vector activity and maximizing resource efficiency. These observations underscore how rootstock growth characteristics interact with management practices and environmental conditions. Vigorous rootstocks such as Swingle may excel in regions with lower disease pressure and abundant resources, while less vigorous rootstocks such as IAC 1600, have advantages in HLB-endemic areas due to their reduced flushing and better adaptation to intensive systems. Also, the smaller canopy volume observed in IAC 1600 may facilitate mechanized or semi-mechanized harvesting, reducing operational costs and increasing harvest efficiency.

### 3.4 Fruit yield and production efficiency

**Figure 4:**
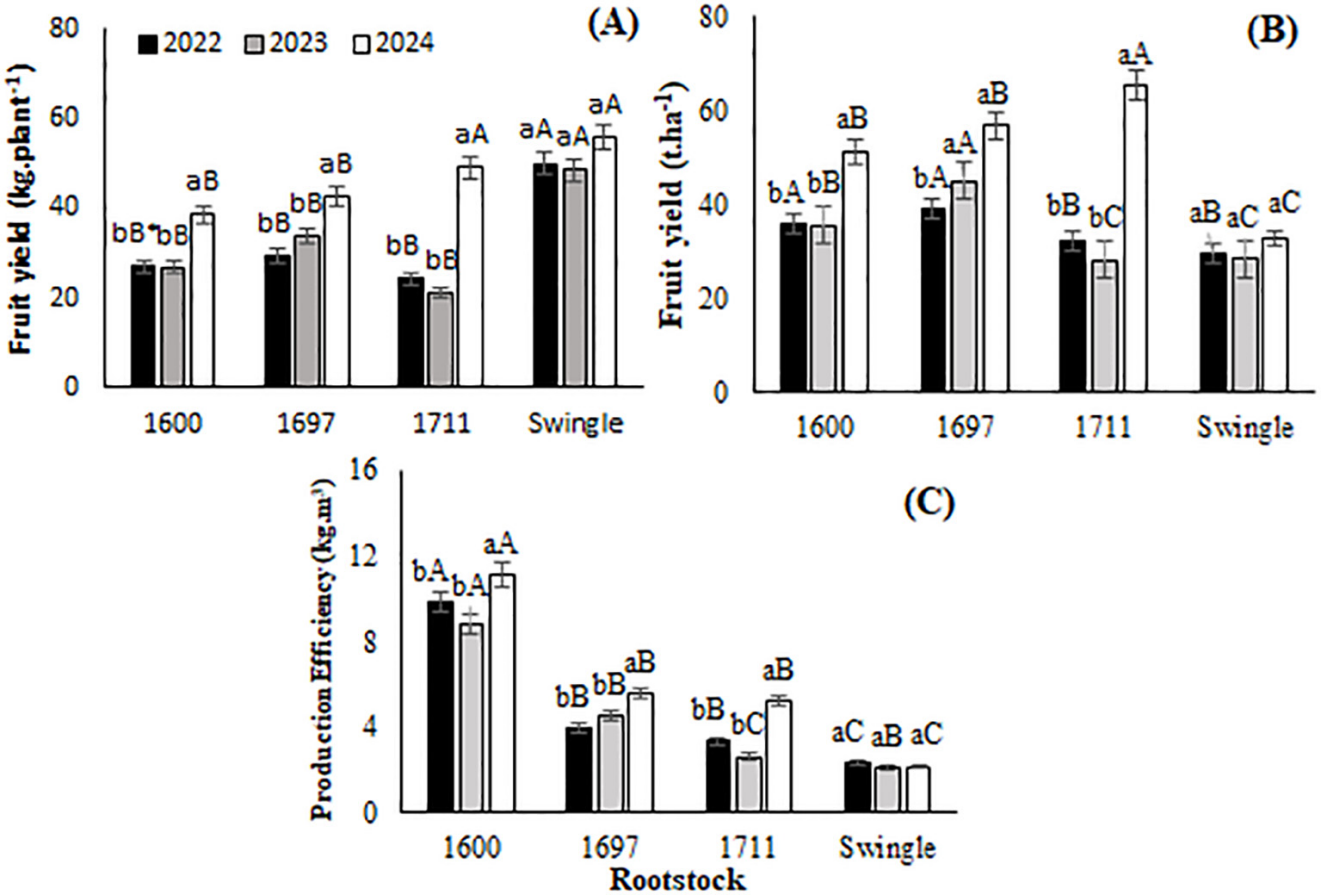
**(A)** Fruit yield (kg.plant^-1^); **(B)** Fruit yield (t.ha^-1^); **(C)** Production efficiency (kg.m^-3^) of Valencia orange grafted onto three citrandarins (IAC 1600, IAC 1697, and IAC 1711) and Swingle citrumelo (Santa Cruz do Rio Pardo, Brazil, 2022-2024). *Means followed by the same lowercase letter within the same rootstock across years, and uppercase letters among rootstocks within the same year do not differ (Tukey, 5%).

Valencia sweet orange grafted onto Swingle citrumelo (**Figure 4A**) exhibited the highest yield per plant in 2022 (50.0 kg·plant^-1^) and 2023 (48.6 kg·plant^-1^), and in 2024, it achieved the highest yield alongside the IAC 1711 combination (55.9 kg·plant^-1^). This consistent performance highlights Swingle’s ability to support high production levels over time. Across the study period, all combinations showed similar yields in 2022 and 2023, with the highest production observed in 2024. In 2024, IAC 1711 induced a higher yield per plant than the other citrandarin combinations.

Yield trends across the rootstock combinations (**Figure 4B**) increased over the years, peaking in 2024. Although Swingle maintained high per-plant yields, the 2023 and 2024 results demonstrated that citrandarin combinations, particularly IAC 1711, outperformed Swingle in total yield.

In terms of production efficiency (**Figure 4C**), IAC 1600 consistently achieved the highest yield per cubic meter of scion volume across all years, followed by IAC 1697 and IAC 1711. In 2024, the IAC 1600 combination reached approximately 11.1 kg·m^-3^, standing out among the combinations. These findings indicate that while Swingle supports high overall production per plant and that citrandarin combinations, such as IAC 1600, offer superior production efficiency, maximizing yield relative to scion volume.

The high and stable productivity observed for the Swingle rootstock across the study period can be attributed to its wider commercial spacing, which accommodates its vigorous growth. However, this vigor may limit its suitability for high-density planting systems, where space is a constraint. In contrast, the superior production efficiency of the IAC 1600 and IAC 1697 combinations reflects their adaptation to high-density planting systems. Girardi et al. (2021) confirmed that vigorous rootstocks, such as Swingle promote robust yields, although production efficiency varies with rootstock characteristics. Domingues et al. (2020) observed that rootstock genotypes significantly influence tree development and production efficiency, with citrandarins demonstrating superior efficiency. Similarly, Vitória et al. (2024) highlighted IAC 1697 for its exceptional production efficiency in Valencia orange, particularly under rainfed conditions, supporting high fruit yields while maintaining an optimal fruit weight-to-scion volume ratio.

Production efficiency is closely linked to rootstock physiology and adaptability to stress conditions. Sampaio et al. (2021) suggested that rootstocks exhibit distinct acclimation mechanisms under drought stress, influencing soil water extraction and overall efficiency. Additionally, Embrapa’s research emphasizes that rootstocks developed for Brazilian conditions, such as citrandarin Indio and San Diego, show high potential for productivity and efficiency in diverse systems, particularly in challenging environments (Costa et al., 2019).

### 3.5 Physicochemical fruit analyses

The physicochemical analyses (**Table 2**) revealed statistically significant differences across all evaluated variables (p<0.05). In the 2021–2022 and 2022–2023 seasons, Valencia sweet orange grafted onto IAC 1711 produced fruit with the highest average weight, whereas Swingle citrumelo induced lighter fruit. However, in the subsequent seasons, Swingle achieved the heaviest fruits, equaling the fruit weight of the IAC 1711 combination in 2022–2023, with averages of 250 and 243g, respectively.

**Table 2:** Physicochemical properties of Valencia orange fruits, including fruit weight, total soluble solids (SS), acidity (TA), SS/TA ratio, juice yield (JY), and technological index (TI), from trees grafted onto 3 citrandarins (IAC 1600, IAC 1697, and IAC 1711) and Swingle citrumelo.

Year	Rootstock	Weight (g)	SS (Brix°)	Acidity (%)	*SS/TA ratio*	JY (%)	TI (kg SST box^-1^)
2021-2022	1600	201.0 b*	11.95 b	0.97 b	12.86 b	54.4 a	2.65 a
	1697	205.5 b	11.80 b	0.85b	13.89 a	56.4 a	2.72 a
	1711	232.4 a	11.04 c	0.95 b	11.69 bc	47.3 b	2.13 b
	Swingle	161.6 c	12.78 a	1.22 a	10.66 c	50.1 b	2.61 a
	CV (%)	14.6	7.9	15.7	11.4	7.9	10.6
2022-2023	1600	194.3 b	11.91 a	0.93 a	12.88 a	53.1 a	2.58 a
	1697	211.1 b	11.76 a	0.91 a	12.93 a	53.3 a	2.56 a
	1711	242.9 a	10.29 b	0.89a	11.56 a	50.4 b	2.12 b
	Swingle	250.0 a	10.35 b	0.90 a	11.70 a	55.6 a	2.35 a
	CV (%)	11.7	7.9	11.9	6.0	14.0	8.9
2023-2024	1600	195.4 c	11.23 a	1.06 a	10.74 ab	52.2 a	2.39 a
	1697	190.1 c	11.61 a	1.09 a	10.67 b	54.8 a	2.60 a
	1711	204.4 b	11.57 a	0.99 a	11.71 a	52.3 a	2.47 a
	Swingle	243.5 a	10.52 b	0.92 a	11.84 a	50.5 b	2.17 b
	CV (%)	11.6	14.5	17.9	15.5	13.3	17.4

*Means followed by the same letter indicate no significant difference (Tukey's test, p<0.05).

Data were collected from a rainfed orchard under Huanglongbing prevalence in Santa Cruz do Rio Pardo, São Paulo, Brazil (2022–2024).

Swingle also recorded the highest SS content during the 2021–2022 season, reaching 12.8°Brix. In the following seasons, however, IAC 1600 and IAC 1697 surpassed Swingle in SS, demonstrating the adaptability of these rootstocks to sustain high-quality attributes. Acidity levels were highest for Swingle in the 2021–2022 season (0.93%), while IAC 1711, IAC 1600, and IAC 1697 showed lower values (0.95, 0.97, and 0.85%, respectively). No significant differences in acidity were observed among the rootstocks in the subsequent seasons.

The ratio, a critical parameter for juice quality, consistently favored IAC 1600 and IAC 1697 across all seasons, indicating a superior flavor balance compared to Swingle and IAC 1711. Juice yield was also highest for IAC 1600 and IAC 1697, exceeding 52% across all seasons and demonstrating their potential for optimized juice extraction. Conversely, the technological index (TI), reflecting industrial processing potential, ranged from 2.12 to 2.60 across all rootstocks, with no significant statistical differences observed.

As shown in in **Table 2**, there were significant differences in fruit weight, juice ratio, and other physicochemical properties among the evaluated rootstocks. Swingle citrumelo produced the heaviest fruits, consistent with findings by Domingues et al. (2020), who evaluated 20 rootstock genotypes, including Swingle citrumelo, citrandarins (IPEACS-239, IPEACS-256), and Rangpur lime. Their study demonstrated that Swingle supports a larger fruit size and robust growth but induces high vigor, which can limit its suitability for high-density planting. Similarly, in our study, Swingle’s high vigor contributed to its superior fruit weight but increased its susceptibility to HLB, a challenge in disease-endemic regions.

Citrandarin hybrids, particularly IAC 1600 and IAC 1697, exhibited higher juice ratios and favorable sugar-to-acid balances, aligning with the findings of Domingues et al. (2020) and Girardi et al. (2021). Domingues et al. (2020) demonstrated that trifoliata hybrids, such as TSKC (Sunki mandarin) × TRFD (Flying Dragon trifoliate orange), enhance juice quality through a higher SS content and juice ratio, even in less vigorous trees. Girardi et al. (2021) further noted that citrandarins such as IAC 1697 promote sweeter juice with higher soluble solids, making them ideal for juice-oriented production systems. In our study, IAC 1697 outperformed Swingle in juice quality metrics, emphasizing its potential for industrial applications.

Under stress conditions, citrandarins demonstrated superior performance. Vitória et al. (2024) evaluated 16 rootstocks, including IAC 1697, IAC 1711, Flying Dragon, and Rangpur lime, and found that IAC 1697 and IAC 1711 maintained a high fruit quality and productivity under rainfed conditions. These results align with our findings, as IAC 1697 and IAC 1711 demonstrated consistent juice ratios and performance under limited water availability. Costa et al. (2019) similarly highlighted the potential of dwarfing citrandarins, such as Sunki mandarin × trifoliate hybrids, for high-density plantings, citing their ability to achieve a high productivity and SS content while maintaining compact growth habits. This mirrors the performance of IAC 1600 in our study, which combined compact growth with efficient resource utilization.

The juice yield showed no significant differences among rootstocks, consistent with findings by Rodrigues et al. (2016), who observed similar results across San Diego, Riverside, and Indio citrandarins. Their study emphasized that juice yield is influenced more by scion genetics and environmental factors than by rootstock choice. Nevertheless, the superior juice quality of citrandarins, particularly IAC 1600 and IAC 1697, positions them as advantageous for industrial processing and juice-focused production systems.

### 3.6 Fruit maturing curve

**Figure 5:**
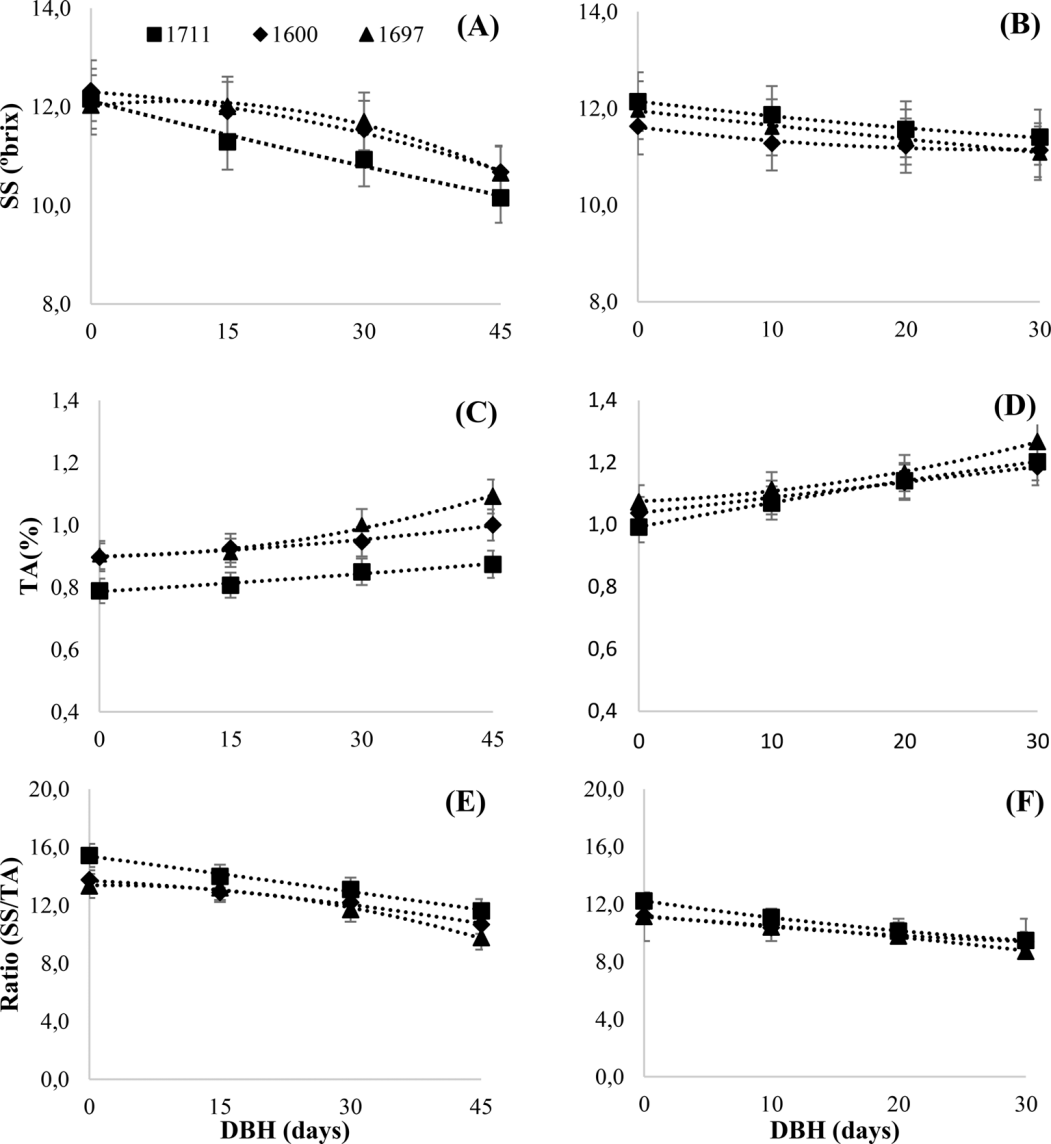
Evaluation of the physicochemical properties of Valencia orange grafted onto three citrandarins (IAC 1600, IAC 1697, and IAC 1711). Panels show: **(A, B)** total soluble solids (SS) in 2023 and 2024; **(C, D)** titratable acidity (TA) in 2023 and 2024; and **(E, F)** ratio in 2023 and 2024. Data was collected over the 45 days before harvest (DBH) in 2023 and 30 DBH in 2024 (Santa Cruz do Rio Pardo, Brazil, 2023-2024).

The polynomial regression equations for total soluble solids (SS), titratable acidity (TA), and SS/TA ratio demonstrated strong fits, with determination coefficients exceeding 0.9 for all tested citrandarins (IAC 1600, IAC 1697, and IAC 1711) (**Supplementary Table S1**; **Figure 5**). An increase in SS accumulation was observed across all rootstock combinations in both years, indicating progressive fruit ripening up to the harvest date. This was particularly evident in the IAC 1711 combination, which reached a SS of 10.16 at 45 DBH and increased to 12.07 by the harvest date.

Environmental factors, such as temperature and precipitation, accounted for 60–70% of the variability in SS content, as previously noted by Albrigo (1992) and supported by more recent studies. Water stress and elevated temperatures contribute to increased SS through water loss in citrus fruit, while excessive rainfall dilutes the juice, reducing SS levels, especially in the days leading up to harvest (Panigrahi et al., 2017).

Climate data from November 2022 to October 2024 corroborate these findings, revealing patterns of fruit acidity reduction during the 2023 and 2024 harvest seasons. The elevated average maximum temperatures, particularly in August and September preceding the October 2023 and September 2024 harvests, ranged between 26 and 28°C and likely accelerated acid degradation. These results align with Marcilla et al. (2006), who observed that higher temperatures near harvest promote faster acid reduction in Valencia oranges. Additionally, in 2024, slightly higher average minimum temperatures (approximately 17.7°C) may have contributed to greater acidity retention, consistent with observations by Russo and Reforgiato Recupero (1984) and Babazadeh-Darjazi et al. (2022) linking temperature variations to acidity accumulation trends in citrus fruits.

The relationship between the SS/TA ratio and DBH was best modeled using positive polynomial regressions for all rootstocks (**Figures 5E, F**). The SS/TA ratio was strongly influenced by rootstock type, with the IAC 1711 combination showing a significant advantage by promoting earlier and achieving the ideal ratio of 12 approximately 30 days earlier than other combinations. This early ripening is particularly beneficial for the juice industry, as it allows for optimized juice quality at a faster rate. These findings align with studies highlighting the role of trifoliate rootstocks and their hybrids in modifying fruit ripening, increasing SS concentration, and reducing acidity during advanced maturation stages (Babazadeh-Darjazi and Jaimand, 2018; Domingues et al., 2020).

### 3.7 Manual harvest efficiency

**Figure 6:**
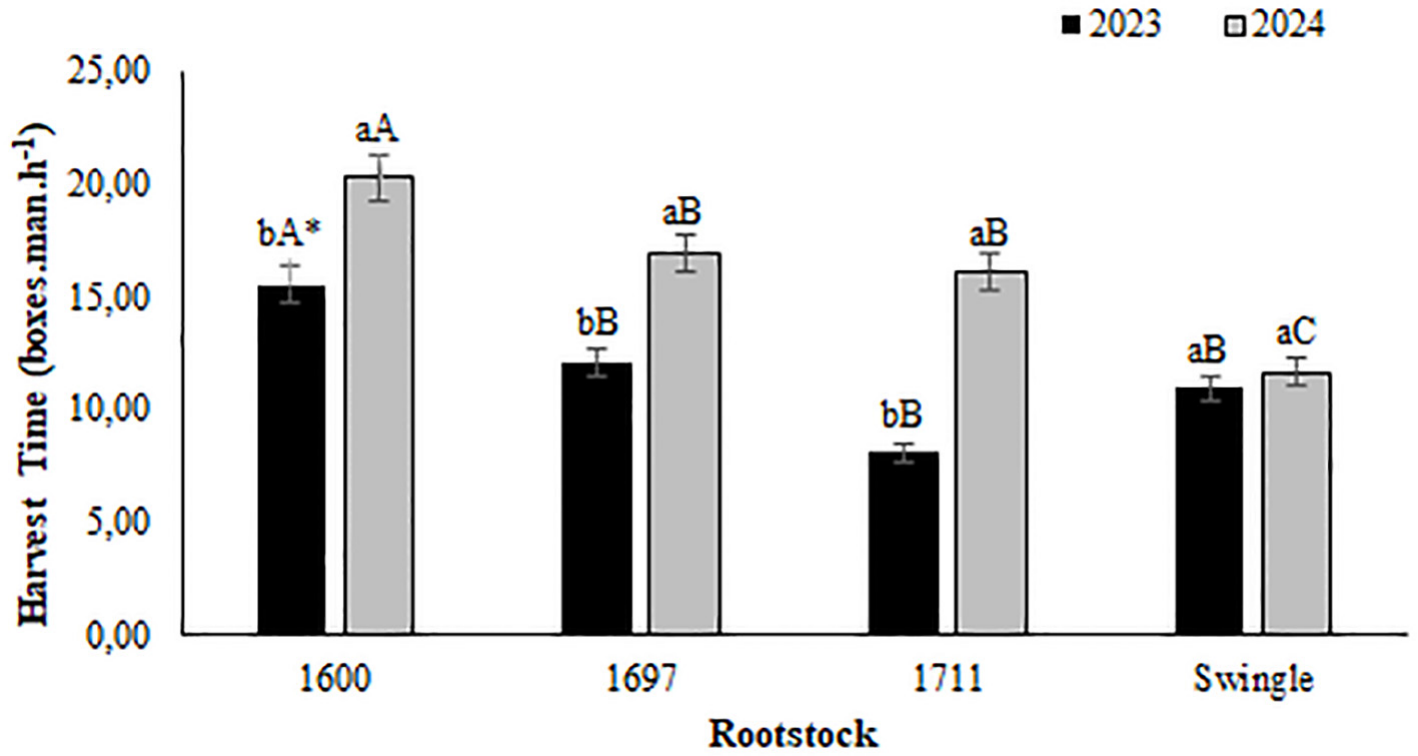
Harvest time (boxes.man.h^−1^) for Valencia orange grafted onto three citrandarins (IAC 1600, IAC1697, and IAC 1711) and Swingle citrumelo (Santa Cruz do Rio Pardo, Brazil, 2023-2024). *Means followed by the same lowercase letter within varieties in the same year and uppercase letters between years within the same variety do not differ (Tukey’s test, 5%).

Analysis of the harvest efficiency (**Figure 6**) revealed that in both 2023 and 2024, the IAC 1600 rootstock combination achieved the highest average values for boxes harvested per hour by a single harvester, with approximately 15.5 and 20.3 boxes.man.hour^-1^, respectively. This superior performance is likely attributable to its smaller canopy size, which facilitates faster harvesting. In 2023, the IAC 1697, IAC 1711, and Swingle combinations showed no significant differences in harvest efficiency. However, by 2024, the Swingle combination recorded the lowest efficiency, with an average of 11.7 boxes.man.hour^-1^. The shorter harvest times associated with the IAC 1600 combination highlight its potential as a preferred choice in systems prioritizing harvest speed and operational efficiency.

These differences in harvest time can be attributed to the intrinsic characteristics of the rootstocks. The Valencia orange-Swingle combination, for instance, is characterized by vigorous growth, leading to larger plants with a more extensive canopy. Although this vigor can enhance the overall yield per plant, it also increases the time required for fruit collection due to the larger canopy volume and less accessible fruit positioning. Conversely, smaller and more compact combinations, such as those with IAC citrandarins, including IAC 1600, promote reduced canopy size and a growth habit conducive to efficient plant management. This compact structure simplifies the harvesting process, as fruit is maintained at a more accessible height, reducing the need for specialized equipment and labor (Rodrigues et al., 2020). These traits make IAC 1600 particularly suitable for high-density planting systems and labor-intensive operations, where harvest efficiency is a critical factor.

### 3.8 Incidence of Huanglongbing

**Figure 7:**
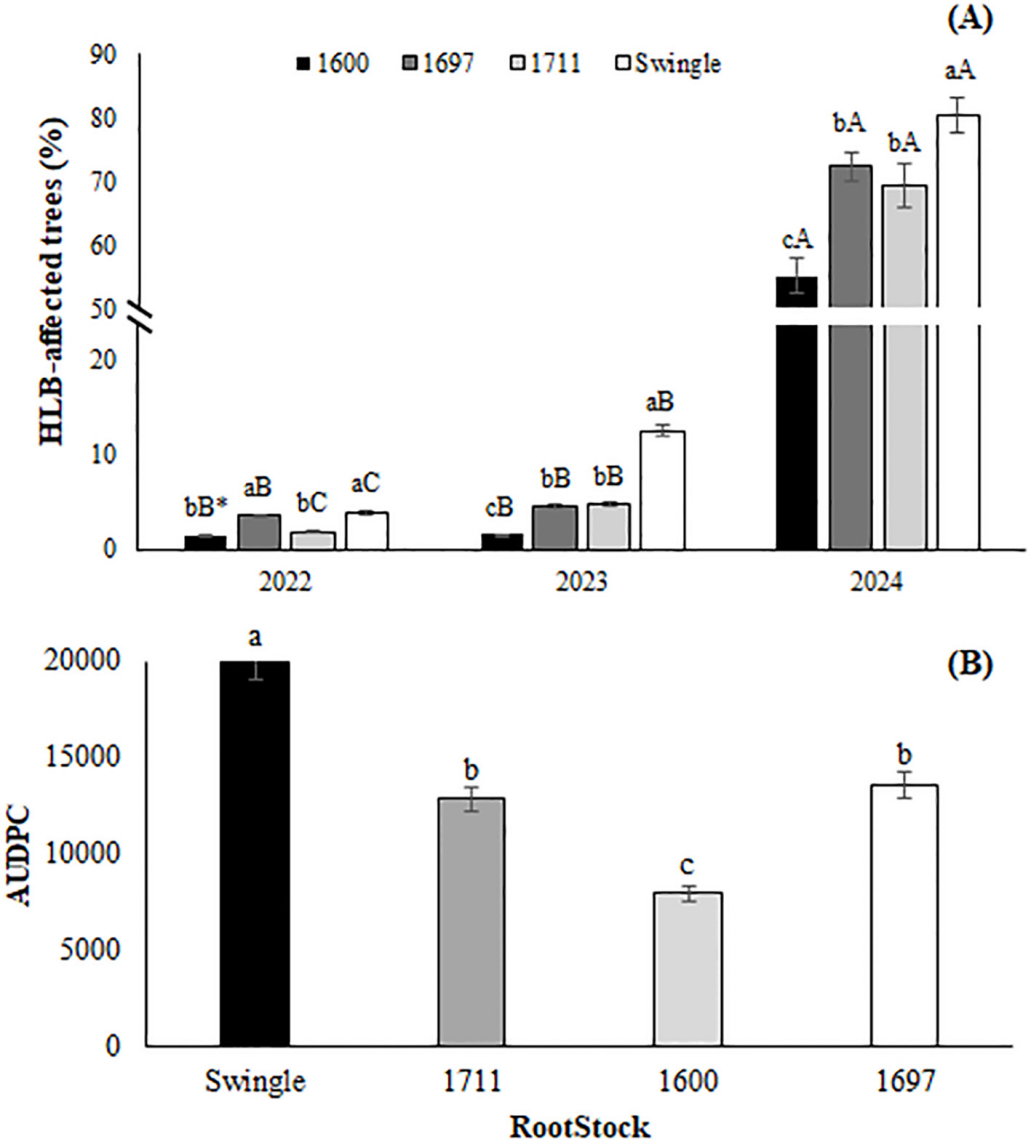
**(A)** HLB-affected trees and **(B)** area under the disease progress curve (AUDPC) in a Valencia orange orchard grafted onto three citrandarins (IAC 1600, IAC 1697, and IAC 1711) and Swingle citrumelo (Santa Cruz do Rio Pardo, 2022–2024). *Means followed by the same lowercase letter within the same rootstock across years and uppercase letters between rootstocks within the same year do not differ (Tukey’s test, 5%).

In 2022, five-year-old Valencia sweet orange trees grafted onto different rootstocks showed a low HLB incidence across all combinations. Swingle citrumelo had the highest incidence at 4.0%, followed by IAC 1697 (3.6%), IAC 1711 (1.9%), and IAC 1600 (1.5%). These findings suggest that Valencia grafted onto IAC 1600 was less susceptible to HLB compared to Swingle citrumelo and IAC 1697. By 2023, six-year-old trees continued to exhibit a similar trend, with Swingle and IAC 1711 showing higher incidences, while IAC 1697 increased to 4.6% and IAC 1600 remained stable at approximately 1.6% (**Figure 7A**).

In 2024, when the trees were 7 years old, a sharp increase in HLB incidence was observed across all rootstocks, likely driven by heightened disease pressure in São Paulo. Swingle citrumelo exhibited the highest incidence (80.4%), followed by IAC 1697 (72.5%), IAC 1711 (69.6%), and IAC 1600 (55.4%). This escalation may be attributed to the growing psyllid population reported by Fundecitrus (2024) and the insufficient rotation of insecticides, which can reduce control effectiveness over time. Recent data showed an 80.4% increase in psyllid captures in 2023 compared to the previous fortnight and a 53.2% rise relative to 2022, emphasizing the critical need for integrated pest management strategies in the region.

Over three years of monitoring, the area under the disease progress curve (AUDPC) was calculated for each rootstock (**Figure 7B**). Swingle citrumelo showed the highest AUDPC values, indicating greater symptom severity, followed by IAC 1711 and IAC 1697 with intermediate values. IAC 1600 consistently displayed the lowest AUDPC, reinforcing its potential as a rootstock for mitigating HLB impact and supporting orchard sustainability.

The vulnerability of citrus scion and rootstock combinations to HLB has been extensively studied, with variable results regarding disease tolerance and severity (Albrecht and Bowman, 2012; Boava et al., 2014; Bowman and McCollum, 2015; Stover et al., 2016; Widyaningsih et al., 2017). Despite these efforts, no scion or rootstock has demonstrated full resistance or immunity to HLB. Swingle citrumelo, consistently reported as HLB-susceptible, aligns with this study’s findings, exhibiting the highest disease incidence (Pompeu Junior and Blumer, 2014).

Citrandarins, such as IAC 1697 and IAC 1600, showed greater tolerance, as evidenced by their lower HLB incidence and AUDPC values. Factors such as controlled canopy volume and reduced flushing dynamics likely contributed to this tolerance, as suggested by Rodrigues et al. (2020). Dwarfing rootstocks, including IAC 1600 and trifoliata Flying Dragon, are associated with reduced shoot flushing and smaller canopies, making them less attractive to psyllids and less conducive to HLB spread. These traits position IAC 1600 as a promising rootstock for regions where HLB is endemic.

The higher HLB incidence of Swingle citrumelo may be tied to its larger canopy volume and frequent flushing, which increase vector attraction. Studies have shown that tree density influences HLB dynamics; and that high-density plantings tend to reduce cumulative HLB incidence, potentially by altering microclimates and limiting vector movement (Moreira et al., 2019; Girardi et al., 2021). These findings highlight the role of cultural practices in disease management, further emphasizing the importance of rootstock selection.

These results underscore the critical role of rootstock choice in managing HLB and sustaining orchard productivity. Continued research into the mechanisms of HLB tolerance and susceptibility across rootstocks is essential for developing integrated management strategies tailored to the challenges of citrus production in HLB-endemic areas.

### 3.9 Principal component analysis

**Figure 8:**
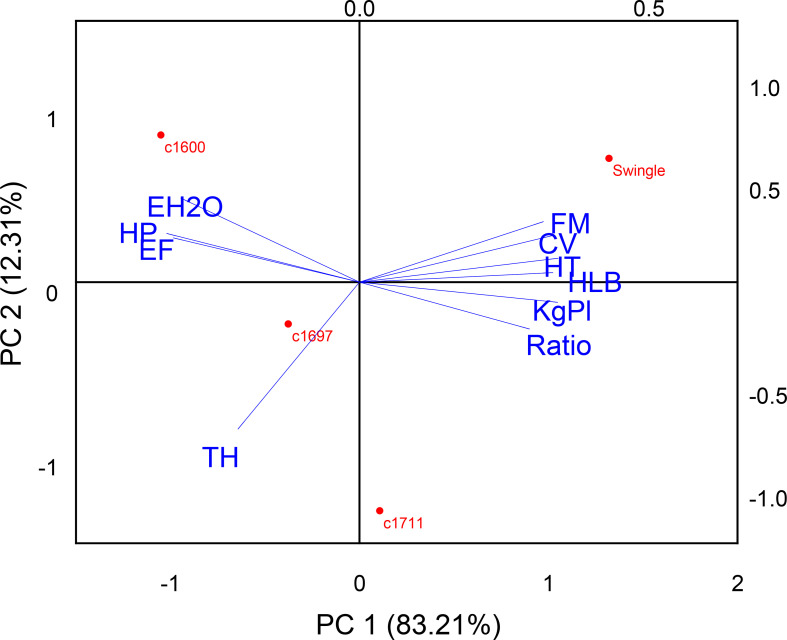
Biplot of principal component analysis (PCA) for Valencia orange grafted onto three citrandarins (IAC 1600, IAC 1697, and IAC 1711) and Swingle citrumelo, showing variables such as canopy volume (CV), water-use efficiency (EH_2_O), production (kg.Pl), productivity (TH), production efficiency (EF), fruit weight (FM), SS/TA ratio (Ratio), harvest time (HT), hydric potential (HP), and HLB.

PC1, accounting for 82.23% of the variance (**Figure 8**), was strongly associated with productivity variables, including canopy volume, fruit weight, total production, and the SS/TA ratio. These variables highlight a positive correlation between high-yield characteristics. In contrast, PC2, explaining 12.31% of the variance, is linked to water-use efficiency traits, such as water-use efficiency and water potential, complementing the productivity-focused attributes of PC1.

Swingle citrumelo is prominently positioned on the positive side of PC1, reflecting its strong correlation with traits, such as larger canopy volume, higher fruit weight, greater total production (kg plant^-1^), and longer harvest time. This rootstock promotes vigorous vegetative growth and enhanced yield, making it well suited for production systems that prioritize output maximization, particularly in environments where water availability is not a constraint. However, its vigorous growth is also associated with a higher HLB incidence, which may limit its suitability in HLB-endemic regions.

In contrast, trees grafted onto IAC 1600 citrandarin were more closely aligned with water-use efficiency and production efficiency variables (kg m^-3^). This rootstock demonstrates superior water-use efficiency, maximizing production per unit of canopy volume while exhibiting a lower incidence of HLB. These traits make IAC 1600 particularly advantageous in water-limited environments, supporting sustainable and resilient production under water-stress conditions.

## 4 Conclusion

The choice of rootstock significantly impacts the productivity and water-use efficiency of sweet orange, which are critical factors for adapting to varying environmental conditions. These findings provide valuable insights for citrus growers in selecting rootstocks that optimize yield and sustainability under water-limited and HLB-endemic conditions. Swingle citrumelo proved effective in maximizing fruit yield, driven by its ability to promote larger canopy volume and per-plant productivity under conditions of adequate water availability. In contrast, citrandarin rootstocks, particularly IAC 1600, exhibited superior water-use efficiency and drought tolerance, making them more suitable for water-limited environments and sustainable orchard management.

The results also highlighted the potential of citrandarin rootstocks in reducing HLB vulnerability, further enhancing their suitability for HLB-endemic regions. Their combination of higher water-use efficiency and lower HLB susceptibility positions them as a viable alternative for promoting orchard sustainability and longevity, especially under drought stress. Rootstock selection should align with management objectives and local conditions, balancing productivity with resource efficiency. These findings offer a robust scientific basis to guide rootstock selection, supporting more productive, resilient, and sustainable citrus cultivation.

## Data Availability

The original contributions presented in the study are included in the article/**Supplementary Material**. Further inquiries can be directed to the corresponding author/s.

## Supplementary material

Supplementary Table 1 Parameters of the polynomial regression equation and coefficient of determination (R²) of SS, TA, and ratio for two seasons of Valencia orange grafted onto 3 Citrandarins (1600, 1697, and 1711) (Santa Cruz do Rio Pardo/SP - 2022-2024).
