# Supplementary material for: Performance of Valencia sweet orange grafted onto dwarfing citrandarins
Source: Front Plant Sci. 2025 Mar 6;16:1530396. doi: 10.3389/fpls.2025.1530396 (PMC11924942; doi:10.3389/fpls.2025.1530396)
Supplement: Supplementary Table 1 — Parameters of the polynomial regression equation and coefficient of determination (R²) of SS, TA, and ratio for two seasons of Valencia orange grafted onto 3 Citrandarins (1600, 1697, and 1711) (Santa Cruz do Rio Pardo/SP - 2022-2024). [file Table1.pdf]

Supplementary Material

Table 1. Parameters of the polynomial regression equation and coefficient of determination (R^2) of SS, TA, and ratio for two seasons of Valencia orange grafted onto 3 Citrandarins (1600, 1697, and 1711) (Santa Cruz do Rio Pardo/SP - 2022-2024)

SS (ºBrix)	Rootstock	Equation	R^2
**2023**	1600	$y = -0{,}0005x^2 - 0{,}0129x + 12{,}299$	0,990
	1697	$y = -0{,}0001x^2 - 0{,}036x + 12{,}087$	0,942
	1711	$y = -0{,}0011x^2 + 0{,}0212x + 12{,}017$	0,992
**2024**	1600	$y = 0{,}0006x^2 - 0{,}0346x + 11{,}612$	0,958
	1697	$y = 5E - 05x^2 - 0{,}0302x + 11{,}948$	0,990
	1711	$y = 0{,}0003x^2 - 0{,}0332x + 12{,}148$	0,996

TA (%)	Rootstock	Equation	R^2
**2023**	1600	$y = 3E - 05x^2 + 0{,}001x + 0{,}8983$	0,985
	1697	$y = 9E - 05x^2 + 0{,}0002x + 0{,}8992$	0,985
	1711	$y = 6E - 06x^2 + 0{,}0017x + 0{,}7861$	0,980
**2024**	1600	$y = -9E - 07x^2 + 0{,}005x + 1{,}0377$	1,000
	1697	$y = 0{,}0002x^2 + 0{,}0017x + 1{,}0754$	0,997
	1711	$y = -4E - 05x^2 + 0{,}0082x + 0{,}9929$	1,000

RATIO (SS/TA)	Rootstock	Equation	R^2
**2023**	1600	$y = -0{,}0007x^2 - 0{,}0342x + 13{,}701$	0,989
	1697	$y = -0{,}002x^2 + 0{,}0081x + 13{,}374$	0,995
	1711	$y = -5E - 05x^2 - 0{,}0799x + 15{,}375$	0,992
**2024**	1600	$y = 0{,}0009x^2 - 0{,}0854x + 11{,}19$	0,997
	1697	$y = 0{,}0012x^2 - 0{,}1279x + 12{,}23$	1,000
	1711	$y = -0{,}0008x^2 - 0{,}0541x + 11{,}118$	0,996
